# Effects and Interactions of Incubation Time and Preplacement Holding Time on Mortality at Placement, Yolk Sac Utilization, Early Feeding Behavior and Broiler Live Performance

**DOI:** 10.3390/ani13243827

**Published:** 2023-12-12

**Authors:** Okan Elibol, Serdar Özlü, Tolga Erkuş, Dinah Nicholson

**Affiliations:** 1 Department of Animal Science, Faculty of Agriculture, Ankara University, Ankara 06110, Turkey; ozlu@ankara.edu.tr; 2 Aviagen Ltd., Newbridge EH28 8SZ, UK; terkus@aviagen.com (T.E.); dnicholson@aviagen.com (D.N.)

**Keywords:** incubation time, preplacement holding time, residual yolk sac, early feeding behavior, mortality, body weight

## Abstract

**Simple Summary:**

The content of nutrients and water in the residual yolk of a newly hatched chick is sufficient to support life for about 3 days under comfortable environmental conditions. In this study, we investigated the impact of preplacement holding times of 6, 24, 48, 60, or 72 h on residual yolk utilisation, the early drive to consume feed and water, growth, and liveability to 7 and 35 d post placement after two different incubation times (504 and 516 h). For robust assessment of small mortality differences, chicks were placed in pens of 160, with 12 replicates per treatment. Longer incubation times resulted in smaller residual yolks at take off and for 24 h afterwards. There were no significant differences in mortality due to holding times between 6 and 60 h, but chicks held for 72 h had a significant increase in mortality at 35 d after placement. In addition, growth to 35 d after placement was depressed in chicks held for 72 h. The trial showed that holding times of up to 60 h after take off had no impact on early mortality or later broiler performance.

**Abstract:**

The effects and interactions of incubation time and chick preplacement holding time on mortality at placement, utilization of yolk sac, crop filling rate, early feeding–drinking behavior, and broiler live performance were investigated. Ross 308 broiler hatching eggs from a 39-week-old flock were set in two identical setters in a commercial hatchery, with the setting time 12 h earlier in one machine. At the end of incubation, chicks were removed from the hatchers at the same time. Thus, the incubation times were either 504 h (normal incubation time (NIT) treatment) or 516 h (longer incubation time (LIT) treatment). After the pull time, chicks from each incubation time group were subjected to either 6, 24, 48, 60, or 72 h preplacement holding times. At placement, chicks were given access to feed and water. In total, 19,200 chicks were randomly assigned to a total of 10 subtreatment groups (2 incubation times × 5 preplacement holding times). Therefore, a total of 1920 chicks were used in each subtreatment group for the grow-out period in a commercial broiler house. For the first week of the experiment, 160 randomly selected as-hatched (not sexed) chicks were placed in 12 replicate floor pens (120 total pens). From the second week of age onward, chicks from two pens were combined into six replicate pens, with 320 chicks per replicate (60 total pens). An interaction was found between incubation time and preplacement holding time for residual yolk sac (RYS) weight (g, %) (*p* < 0.001). RYS weight was greater at pull time and at 6 and 24 h of preplacement holding in the NIT treatment compared to the LIT treatment, while differences were no longer evident at 48–72 h. The lowest percentage of chicks with full crops and eating activity was observed in the shortest preplacement holding time (6 h) group at 3 h after placement. As expected, the initial BW at placement clearly decreased with increasing duration after the pull time (*p* < 0.05), with the highest and lowest weights found in the 6 and 72 h holding time treatments, respectively. This BW difference was still evident at 35 d after placement and chicks held for the longest period after the pull time (72 h) showed the lowest BW (*p* < 0.001). However, there was no significant difference between the 6 and 60 h preplacement holding times. Mortality during the first 7 d after placement increased only when the preplacement holding time was extended to 72 h (*p* = 0.031). Similarly to the 7 d results, chicks held for 72 h exhibited higher 0–35 day mortality compared to those held for 6 or 24 h (*p* = 0.028). Neither BW nor mortality was affected by incubation time treatment at 35 d after placement (*p* > 0.05). It can be concluded that there were no significant differences in average BW and mortality, up to and including a 60 h holding time under thermal comfort conditions, but a 72 h preplacement holding time resulted in final BW and mortality being negatively affected. In addition, LIT tended to have a beneficial effect on BW and mortality compared to NIT when the preplacement holding time was shorter (6–24 h) but had a negative effect for extended holding times (48–72 h).

## 1. Introduction

Under commercial conditions, chicks commonly hatch within a 24 to 36 h time period and a total of incubation time of approximately 504 h, which is considered optimal for the complete hatching of incubated eggs [1]. Incubation time is known to be mainly influenced by strain, parental age, egg size, storage time, and eggshell temperature before and during incubation [2,3,4,5,6,7]. Early collection of chicks from the hatcher tends to increase the percentage of second quality chicks with unhealed navels, while delaying the chick collection leads to a higher percentage of dehydrated chicks. After removal from the hatcher, access to feed and water will usually be delayed while chicks are selected, sexed, vaccinated, and packed to be transported to the farm. The interval between take off and arrival on farm is usually 6 h or less for broiler chicks, but for high-generation breeding stocks, which are often farmed and hatched in remote locations to optimize disease control and biosecurity, the interval between pulling and placement times can be up to 60 h [8]. Nevertheless, the energy content of fat in the residual yolk of the newly hatched chick is sufficient to meet chick requirements for about 3 d under optimum thermal conditions [9].

It has been reported that longer holding periods after hatching, either in the hatcher or after pull time prior to placement in the broiler house, adversely affected early chick quality [10], increased early mortality [11,12,13], and reduced growth [3,13,14,15,16]. Several other studies, where chicks were similarly held for 24 h or longer after hatching under suitable conditions, recorded no evidence of clinical dehydration of the chicks nor any effect on live broiler performance [1,17,18,19,20,21,22]. In all the above studies, pen sizes were small, with relatively few replicates. As a result, mortality differences, in particular, were unlikely to be statistically significant. On the other hand, in a recent study [23], a high number of chicks (19,200 chicks) with larger pens (160 chicks/pen) were used in a commercial broiler house. The study showed that while mortality was not affected by preplacement holding times up to and including 60 h after take off under thermal comfort conditions, holding for a further 12 h to 72 h increased mortality to 7 d of age. Although the increase was numerically small, it was statistically significant (*p* = 0.02).

It has been proposed by the EFSA (European Food Safety Authority) Panel on Animal Health and Welfare (AHAW) [24] that in order to improve bird welfare, the maximum time between hatching and first access to feed and water (including time spent in the hatchery, holding time, loading, transport, and unloading time) should not exceed 48 h. However, the panel accepts that more research is needed to assess the maximum time before delayed access to feed and water becomes detrimental for day-old chicks.

In the current study, a large number of chicks (19,200) were raised to investigate the effects and interactions of incubation time and preplacement holding times up to and including 72 h after the pull time on mortality at placement, yolk sac utilization, crop filling rate, early feeding–drinking behavior, and BW and mortality at 35 d after placement. The results can provide guidance on chick transportation for commercial companies and farmers.

## 2. Materials and Methods

All procedures in the current study were approved by the Animal Ethics Committee of the Poultry Research Institute, Ankara (2021/01).

### 2.1. Hatching Eggs and Incubation

Hatching eggs were obtained from a commercial broiler breeder flock of Ross 308 at 39 wk of age and stored for 3 d at 16 °C and 75% relative humidity (RH). After storage, a total of 38,400 hatching eggs were set equally and randomly in two identical setters (Petersime, Zulte, Belgium) in a commercial hatchery (Beypiliç Inc., Bolu, Türkiye). Half of the total eggs (19,200 eggs) were set 12 h earlier than the other half (19,200 eggs). The space remaining in each setter (with a maximum capacity of 57,600 eggs) was filled with hatching eggs that were not part of the experiment, to ensure uniform airflow across the eggs. The filler eggs were from two different flocks of similar age and hatchability to the trial eggs.

Eggs from both setters were prewarmed at 28 °C for 6 h before incubation. Then, a standard single-stage incubation program was used with a gradually decreasing machine set-point temperature of 38.1 °C at embryonic day (E) 1 of incubation to 37.0 °C at E19. RH was 70% during the first 10 d of incubation (minimum ventilation) and then ventilated to maintain 40% RH until E19. Eggs were turned 90° on an hourly basis until E19, at which time they were transferred to hatching baskets and placed in hatchers. The hatcher temperature was initially set to 37.2 °C and gradually decreased to 36.4 °C over three days (until E21).

The experiment was designed to test the impact of incubation time on the livability and performance of chicks after various holding times between pull and placement on the farm. To ensure that the chick holding and brooding conditions were identical, the eggs for the longer incubation time (LIT) treatment (516 h) were set in a separate setter 12 h earlier than those for the normal holding time (NIT) treatment (504 h). Both groups spent 456 h in the setter, then on day 19 were transferred to two separate hatchers, where the LIT treatment eggs spent 12 h longer than the NIT. The last 12 h in the hatcher for the LIT treatment was programmed to deliver 36.4 ± 0.4 °C and 53 ± 2% RH, to achieve a chick vent temperature of 39.4–40.5 °C.

### 2.2. Chick Management and Experimental Design

At the pull time, chicks were visually sorted according to commercial hatchery standards: weak chicks or those with physical abnormalities, unhealed navels, or red hocks considered unsaleable were culled. In total, 88.0% of the chicks hatched in the NIT treatment, and 88.8% of the chicks hatched in the LIT treatment. The percentage of second-grade chicks was 0.67% and 0.47% in the NIT and LIT treatments, respectively.

First quality chicks were counted, then spray vaccinated against infectious bronchitis and Newcastle disease. The boxes of chicks (80 chicks per box) were packed into an unlighted climate-controlled truck (H90, Heering, Vaassen, Holland) and then transported to the commercial broiler house for 2 h. Chicks were subsequently held in the running truck at 25.7 ± 0.3 °C until placement.

The boxes of chicks were randomly allocated into 5 different preplacement holding times, including 6, 24, 48, 60, and 72 h after pull time in each incubation time treatment (NIT and LIT). In total, 19,200 chicks were randomly assigned to 10 subtreatment groups (2 incubation times × 5 preplacement holding times); therefore, a total of 1920 chicks were used in each subtreatment group for the grow-out period. For the first week of the experiment, 160 randomly selected as-hatched (not sexed) chicks belonging to one of the 10 subtreatment groups were placed in each of 12 replicate floor pens (120 total pens). From the second week of age onward, chicks from two pens were combined into 6 replicate pens (60 total pens) with 320 chicks per replicate.

### 2.3. Grow-out Housing and Management

The birds were grown from placement to 35 days in a commercial broiler house, which was preheated for 24 h before the first chicks were placed, to deliver a uniform and steady litter temperature of 30 °C. From placement, the ambient temperature was gradually decreased from approximately 32 °C to 20 °C at the end of the experimental period (d 35). Chicks received 23 h of light (23 L:1 D) during the first 10 d. From Day 11 until the end of the study, the chicks were given 4 h of darkness between 23:00 h and 03:00 h.

Chicks were housed in floor pens (1.50 × 2.75 m) containing new wood litter shavings until 7 d from the day of placement in each group. From the second week of age onward, chicks from two pens were moved into one large pen in each subtreatment group, which measured 1.5 m × 12 m (18 m^2^) and kept under uniform management conditions throughout the study. The initial chick density in each of the pens was approximately 0.026 m^2^ per bird. From the second week onward, the chick density in each of the pens was 0.056 m^2^ per bird. Water was provided via two or four nipple lines with 18 or 36 nipples per pen during the first and from the second week of age onward throughout the remainder of the study, respectively. Feed was accessed from two (used for the first 7 d) or eight (used from 8 to 35 d) 33 cm diameter pan feeders running along the midline of each pen. Additional feed was offered on paper in all pens for the first 4 d after placement.

Starter and grower diets were fed from 0 to 10 d and 11 to 24 d, respectively. The finisher diet was fed from 25 to 35 d. Starter feed was produced in crumble form, while the other feeds were manufactured in pellet form (3.5 mm in diameter). The diets for each feeding period were formulated to meet or exceed the demands of broiler chickens according to the recommendations of the breeder company [25] until d 35.

### 2.4. Measurements

All on-farm measurements were performed on a defined number of days after chicks were placed into the pens with free access to feed and water. The time elapsed since the chicks were removed from the hatcher thus varied by up to 3 d.

#### 2.4.1. Vent Temperature

Chick body temperature was determined by recording the vent temperature of 100 randomly selected chicks (10 chicks/box/treatment) using an infrared digital thermometer (IRT 4520, Thermoscan, Braun GmbH, Kronberg, Germany) at pull time and chick placement times.

#### 2.4.2. Mortality at Placement Time (DOA)

All chick boxes were opened, and dead chicks (dead on arrival, DOA) were recorded to determine the percentage of mortality relative to the total chicks at each placement time.

#### 2.4.3. Residual Yolk Sac (RYS) Weight and Yolk-Free Body Mass (YFBM)

RYS and YFBM weights were measured at pull time (80 randomly selected chicks from each incubation time treatment) and at the end of each of the 5 holding periods (40 randomly selected chicks) in each incubation time treatment group. The yolk-free body mass (YFBM) was calculated as the chick weight minus the residual yolk weight. In the present study, residual yolk sac weights were determined in a total of 800 chicks. In addition, the RYS weight was also recorded to determine the yolk sac utilization of fasted and non-fasted chicks. Further information concerning the measurement of RYS and YFBM is provided by Özlü et al. [23].

#### 2.4.4. Crop Filling and Feeding-Drinking Behavior

Crop filling was examined in 30 randomly selected chicks from each pen at 3 h after placement. Chick feeding and drinking behavior were determined within 1, 3, and 8 h of placement within pen and all chicks were counted. The procedures of the crop filling and feeding–drinking behavior were as previously described by Özlü et al. [23].

#### 2.4.5. Live Broiler Performance (BW and Mortality)

Body weights (BWs) were recorded at placement and 7 d of age by bulk weighing in each pen. At 35 d from the day of placement, a random sample of 50 chickens (25 female and 25 male chickens) per pen was individually weighed in each group. Weighing times were organized so that each treatment had 35 full days of feed availability. Mortality in each pen was recorded six times a day throughout the study.

### 2.5. Statistical Analysis

Five separate analyses consisting of Fisher’s exact test (mortality at placement time), a one-way ANOVA (RYS weights between groups with differing access to feed and water (fasted/no-fasted) at each sampling time), and two or three factorial arrangement (2 × 5 or 2 × 5 × 2 factorial designs) were undertaken. Prior the analysis of ANOVA, normal distribution of the data was checked with a Kolmogorov–Simirnov test, and the homogeneity of the group variances was assessed with a Levene test.

Effects of incubation time treatment (NIT of LIT), preplacement holding time (6, 24, 48, 60 or 72 h), gender, and access to feed and water (fasted or no-fasted) on collected data were analyzed using the general linear models procedure of SAS.

The RYS weight (g, %), YFBM, crop filling, feeding behavior, BW, and chick mortality data were analyzed as appropriate for a 2 × 5 factorial arrangement of treatments. For RYS weight and YFBM, the data were collected per individual, but the crop filling and feeding behaviour percentages and chick mortality were calculated for 12 replicate pens in all sub-treatments. The two factors were incubation time treatment and preplacement holding time. Sex was included as a main factor for analysis of the BW at day 35, so the analysis was performed with a 2 × 5 × 2 factorial arrangement.

Additionally, data of RYS weights were analyzed as appropriate for a 2 × 5 × 2 factorial arrangement of treatments. The three factors were incubation time treatment, preplacement holding time, and access to feed and water.

All data were expressed as least square means, and Duncan’s multiple range tests was performed for the mean comparison.

All statistical analyses were performed with SAS version 9.1 (SAS Institute Inc., Cary, NC, USA).

## 3. Results and Discussion

### 3.1. Vent Temperature

Chick body temperature depends on surrounding environmental temperature and affects the quality of day-old chicks and performance of chicks later in life [26,27]. For these reasons, cold and heat stress have been selected as having highly relevant welfare consequences for day-old chicks [24].

Chick body temperature is determined either with an infrared ear thermometer placed on the cloaca (vent) or by measuring deep body temperature by inserting a thermometer in the cloaca (rectal). Chick vent temperature is highly correlated with deep body (rectal) temperature [28], although it was found to be 0.5 °C lower than rectal temperature [29]. For a day-old chick to be comfortable, it is recommended that the vent temperature be in the range of 39.4–40.6 °C [8] or the cloacal temperature be in the range of 40.0–41.0 °C [24].

In the present study, chick body temperature was easily and confidently determined by recording the vent temperature. No significant differences in vent temperature were found between the pull time and the preplacement holding times, and the average vent temperature was kept in the ideal range (39.7 ± 0.65 °C), as shown in Table 1.

### 3.2. Mortality at Placement Time (DOA)

The percentage of cumulative mortality was higher in the 72 h preplacement holding time (0.107%) than in the 6 h (0.005%), 24 h (0.024%), and 48 h (0.032%) holding times (*p* < 0.001, *p* = 0.014, and *p* = 0.021, respectively). Furthermore, the cumulative mortality at 60 h (0.057%) was also significantly higher than that at 6 h (*p* = 0.005). However, this was due to higher mortality for the LIT (0.075%) than for the NIT (0.039%) treatment (Table 2).

DOA is also a relevant indicator for the assessment of chick welfare during the preplacement holding time [30,31]. However, very little is known in the literature about the specific DOA of day-old chicks. In a field study from Yerpes et al. [32], the mean chick mortality during transport was 0.055%. The AHAW Panel concluded that DOA should not exceed 0.10% [24]. Recently, Özlü et al. [23] conducted an experiment similar to the current study and reported that mortality at placement was not affected up to 60 h of holding time. However, in the study of Özlü et al. [23], when the holding time was extended to 72 h (0.244%), mortality was significantly higher than in the 6 h (0.020%) and 24 h (0.039%) preplacement holding time groups (*p* < 0.05). Similarly, in the current study, the highest mortality was observed for the 72 h holding time group, although it was very low (0.107%). In addition, LIT increased mortality at placement when the preplacement holding time was extended (60–72 h) (Table 2). Therefore, to reduce mortality at placement, chicks held for longer preplacement holding times should not be exposed to long incubation times.

### 3.3. Yolk-Free Body Mass (YFBM) and Residual Yolk Weight (RYS)

In the current study, at placement, the chicks in the LIT group, with an incubation time of 516 h, had a lower YFBM and less residual yolk (*p* < 0.001) than those in the NIT treatment (504 h). In addition, the YFBM was affected by the preplacement holding time and decreased with increasing preplacement holding time. From the pull time to the 24, 48, 60, and 72 h holding times, YFBM was reduced by 2.7%, 4.7%, 6.1%, and 10.9%, respectively (Table 3). This finding was consistent with Özlü et al. [23], who found that the percentage of YFBM decreased more between 60 and 72 h.

As expected, the absolute and relative RYS weights (g, %) decreased with increasing holding period after the pull time in each incubation time treatment. However, an interaction was found between incubation time and preplacement holding time for RYS weight (g, %) (*p* < 0.001). RYS weight was greater at pull time and at the 6 and 24 h holding times in the NIT treatment than in the LIT treatment, whereas these differences were no longer evident at 48 h and longer holding times.

It was assumed in the EFSA [33] that modern genetic lines may deplete their reserves more quickly due to the higher metabolic rates associated with faster growth. A higher metabolic rate during incubation will lead to a lower residual yolk weight and energy reserve for the hatchling, which might affect post-hatch development and performance. In contrast to the EFSA [33], Aviagen [8] conducted a trial to compare the rate of yolk utilization in male line chicks from a 1972 genetic control line and a current equivalent line from 2017. The rate at which the residual yolk was depleted was very similar (83% of the residual yolk) in both the control lines and their modern counterparts at 72 h post chick takeoff. In the current study, similarly to Aviagen [8], 85% of the residual yolk was utilized at the 72 h preplacement holding time. In addition, in a recent review on yolk sac utilization in poultry, Van der Wagt et al. [34] reported that genetic progress and improved management and incubation conditions have led to limited effects on yolk utilization efficiency and embryonic metabolic heat production.

In the current study, in which the maximum preplacement holding time was 72 h after pull time, the absorption of the yolk sac was not affected by fasting (Table 4). This finding was consistent with the findings of recent studies that did not find differences in yolk utilization or residual yolk weights between immediate and delayed post-hatch feed intake up to 72 h [21,23,35,36,37,38].

### 3.4. Crop Fill Progression

In the present study, interactions between the preplacement holding time and incubation time were found for the percentage of empty and full, rounded crops (water and feed) at 3 h after placement (Table 5). The percentage of empty crops was significantly higher only at the 6 h holding time in the NIT treatment compared to all other combinations (*p* < 0.001). On the other hand, the percentage of birds with full crops increased with increasing duration after the pull time, and except for the 6 h holding time, all preplacement holding time groups were over the target crop filling recommendations (75–80%) by Aviagen [39] at 3 h after placement in both incubation time treatments. Moreover, the percentage of chicks with full crops in the NIT treatment (54.4%) was significantly lower than that in the LIT treatment (68.3%) at 3 h after placement (*p* < 0.001) in the 6 h preplacement holding time group. These findings were consistent with those of Boyner et al. [40] and Özlü et al. [23], who found that the full crop percentage increased when chicks were held for an additional period of time before placement.

### 3.5. Behavior Observations

In the current study, feeding and drinking behaviors were observed in each treatment at 1, 3, and 8 h after placement. No interactions between preplacement holding time and incubation time were found for chick behaviors at any observation time after placement. Incubation time had no impact on chick behaviors (eating–drinking) after placement (*p* > 0.05) (Table 6). However, chicks with the shortest (6 h) preplacement holding time had a lower percentage of feed-seeking activity than the other holding time groups at 1, 3, and 8 h after placement (*p* < 0.001). This was confirmed by the crop fill progression, in which the lowest percentage of chicks with full, rounded crops was found in the 6 h preplacement holding time (Table 5). However, these changes in crop fill and early eating and drinking activity did not lead to comparable improvements in 7- and 35-d BW and mortality. Therefore, crop fill and eating activity at the beginning of production might not be good indicators of animal well-being and health, which was also confirmed by Özlü et al. [23].

### 3.6. Body Weight

In the current study, chicks in the LIT treatment weighed 4.06% less than those in the NIT treatment at placement (*p* < 0.001). However, this advantage for the NIT treatment was no longer evident for BW at 35 d after placement (Table 7). As expected, males exhibited greater BW than females at 35 d (*p* < 0.001), and no interaction effects were observed between sex and other factors (*p* > 0.05).

In the current study, chick weight at placement was significantly reduced by duration after the pull time (*p* < 0.05), and the highest and lowest body weights (BWs) were found in the 6 (44.8 g) and 72 (38.0 g) h preplacement holding time groups, respectively (*p* < 0.001). There was no interaction between the preplacement holding time and incubation time for BW at placement. However, an interaction was found between the two treatments for BW at 7 d after placement. The BW differences between the NIT and LIT treatments were −3.2, −1.1, +3.4, +5.8, and +7.9 g at 6, 24, 48, 60, and 72 h, respectively, at 7 d after placement, which was an advantage in the LIT treatment for short holding times (6–24 h) but a negative effect for prolonged holding times (48–72 h).

At 35 d after placement, chicks held for the longest period after the pull time (72 h) showed the lowest BW (*p* < 0.01). In contrast to placement time, no significant differences occurred for the 6 to 60 h preplacement holding times, and the highest BW was found in the 24 h holding time at the end of the trial. Numerous studies have shown that BW was negatively affected by longer post-hatch holding periods in the early period of growing, but no differences were observed at the end of grow-out period [17,41,42,43,44,45,46,47].

It has been reported that broilers were able to compensate for 36–54 h of feed deprivation when they were able to access feed for a similar period of time at 35 d [20,21,48] or 41 d [49]. In a similar study by Cardeal et al. [50], chicks were subjected to 3, 24, 48, and 72 h preplacement holding times after pulling from the hatchery. When the day of placement was considered the first day, fasting up to 72 h did not have any negative effect on BW, FCR, and mortality at 39 d. In the current study, BW at 35 d was similar in the 6 h and 60 h preplacement holding time groups (2179 vs. 2148 g), whereas 72 h holding time (2098 g) without feed and water access after pull time could not compensate for the loss in growth compared with the other holding time groups at 35 d after placement. Although there was no interaction between the preplacement holding time and incubation time for BW at 35 d after placement, with a similar trend at 7 d, the LIT treatment that positively affected BW for the short holding times was not favorable for longer holding times. Furthermore, at 7 d after placement, the BW difference between 6 and 72 h in the NIT treatment was 10.3 g, whereas this difference was 21.4 g in the LIT treatment. Similarly, BW differences were two times higher in the LIT than in the NIT treatment (111 g vs. 50 g) between 6 and 72 h holding times at 35 d after placement (Table 7).

### 3.7. Mortality

In the current study, incubation time had no impact on mortality at 7 and 35 d after placement. However, mortality was affected by preplacement holding time and reached 1.82% in the 72 h group at 7 d, which was significantly higher than that in the other preplacement holding time groups (*p* = 0.031). Özlü et al. [23] also reported similar findings, where holding chicks for 72 h increased 7 d mortality more than those held for 6–60 h preplacement. Similarly, a large number of chicks (19,200) were reared in the current study, and mortality, a direct indicator of flock health, was not affected by holding times up to and including 60 h at 35 d. However, similarly to for 7 d, holding for a further 12 to 72 h increased mortality (6.15%) compared to the 6 h (4.85%) and 24 h (4.22%) preplacement holding time groups (*p* = 0.028; Table 8).

De Jong et al. [51] carried out a detailed meta-analysis of data from multiple peer-reviewed published experiments examining the impact of delayed feeding on broiler performance and welfare traits. While a total of 84 experiments were initially identified as possible candidates for meta-analysis, in categorizing the data prior to analysis, they found that while some of the experiments started timing in the hatcher, soon after individual chicks emerged from the shell (Category 1), others started when the hatcher was opened to remove all the chicks (Category 2), while for a final group the experiment only started when the chicks were placed in pens at the experimental farm, an unknown number of hours after both emergence and pull (Category 3). Of the 84 datasets originally identified, 42 supplied body weight data suitable for analysis, whereas only 12 of them reported usable mortality data, to 7 days, 42 days, or both. The meta-analysis suggested that a post hatch delay of 48 h or longer in offering feed increased total mortality to 42 days. However, the 12 datasets included had equal numbers falling into each of the start time categories, with half of them also having either small pen sizes or low numbers of replicates. In addition, some of the delayed-fed chicks were held in boxes in the chicken house. Given that optimal house brooding temperatures are some 6–8 °C warmer than those normally used in chick delivery vehicles, these birds almost certainly overheated, which could be expected to increase early mortality [27]. On the other hand, in much larger scale experiments, Dişa et al. [49] examined the interaction effect of hatching time and pull time on broiler live performance. Chicks were held in the hatcher for 7, 17, 26, 31, 41, or 50 h after hatching, and the highest mortality at 41 d was found in chicks that were held in the hatcher for the shortest time (7 h) (*p* < 0.001). In the current study, although there was no interaction (*p* > 0.05) between incubation time and preplacement holding time on mortality at 35 d (Table 8), mortality differences between NIT and LIT treatments were -0.93, -0.32, +0.53, +0.42, and +1.04% at 6, 24, 48, 60, and 72 h, respectively. This was apparently beneficial for mortality in the LIT treatment, as indicated by BW, at short holding times (6–24 h) but detrimental at long holding times (48–72 h). In addition, the mortality difference between 6 and 72 h in the NIT treatment was 0.5%, whereas this difference was 2.0% in the LIT treatment at 7 d after placement. Similarly, mortality differences were 0.3 and 2.3% in the NIT and LIT treatments, respectively, at 35 d (Table 8), which was consistent with the percentage of mortality at placement (Table 2).

## 4. Conclusions

In the present study, using a total of 19,200 chicks, preplacement holding times up to and including 60 h after pull time under optimum thermal conditions had no effect on BW and mortality at 35 d of age but reduced BW and increased the mortality when the preplacement holding time was extended to 72 h. In addition, the longer incubation time (LIT-516 h) tended to have a beneficial effect on BW and mortality when the preplacement holding time was shorter (6–24 h), but these parameters were negatively affected with extended holding times (48–72 h) compared to the normal incubation time (NIT-504 h).

## Figures and Tables

**Table 1:** Vent temperature of chicks.

Treatment	Vent Temperature ^1^
	------------- (°C) --------------
Incubation time (h)	
NIT (504)	39.73
LIT (516)	39.69
SEM	0.029
Preplacement holding time (h)	
0	39.61
6	39.72
24	39.67
48	39.78
60	39.73
72	39.74
SEM	0.060
*p* values	
Incubation time	0.326
Preplacement holding time	0.078
Incubation time × Preplacement holding time	0.121

^1^ *n* = 100 for all sub treatments; No significant differences in vent temperature were found between the pull time and the preplacement holding times.

**Table 2:** Cumulative mortality during preplacement holding time.

Incubation Time (h)	Preplacement Holding Time (h)				
	0–6	0–24	0–48	0–60	0–72
	------------------------------------------ (%) ------------------------------------------				
NIT (504)	0.010 ^c^	0.022 ^bc^	0.039 ^bc^	0.039 ^bc^	0.089 ^a^
LIT (516)	0.000 ^c^	0.025 ^bc^	0.025 ^bc^	0.075 ^a^	0.125 ^a^
Combined	0.005 ^c^	0.024 ^bc^	0.032 ^bc^	0.057 ^ab^	0.107 ^a^

^a–c^ Mortality percentages followed by different letters differ significantly (*p* < 0.05).

**Table 3:** Effects of incubation time and preplacement holding time on YFBM and RYS (g and percentage) of chicks.

Treatment		YFBM		Residual Yolk Sac (g and Percentage)	
		g		g	%
Incubation time					
NIT (504)		39.83 ^A^		2.86 ^A^	6.38 ^A^
LIT (516)		38.83 ^B^		2.22 ^B^	5.15 ^B^
SEM		0.155		0.069	0.140
Preplacement holding time (h)					
0		41.09 ^a^		4.73 ^a^	10.20 ^a^
6		40.57 ^ab^		4.47 ^a^	9.85 ^a^
24		39.98 ^b^		2.72 ^b^	6.32 ^b^
48		39.14 ^c^		1.45 ^c^	3.53 ^c^
60		38.57 ^c^		1.15 ^c^	2.83 ^c^
72		36.61 ^d^		0.71 ^d^	1.86 ^d^
SEM		0.261		0.116	0.235
Incubation time × Preplacement holding time (h)					
NIT (504)	0	41.56		5.34 ^Aa^	11.30 ^Aa^
	6	41.03		5.13 ^Aa^	11.10 ^Aa^
	24	40.50		3.06 ^Ab^	7.02 ^Ab^
	48	39.48		1.59 ^Ac^	3.83 ^Ac^
	60	39.10		1.27 ^Acd^	3.10 ^Ac^
	72	37.27		0.76 ^Ad^	1.94 ^Ad^
LIT (516)	0	40.63		4.12 ^Ba^	9.11 ^Ba^
	6	40.10		3.81 ^Ba^	8.60 ^Ba^
	24	39.45		2.38 ^Bb^	5.62 ^Bb^
	48	38.79		1.31 ^Ac^	3.22 ^Acd^
	60	38.04		1.02 ^Ac^	2.56 ^Ade^
	72	35.94		0.66 ^Ac^	1.78 ^Ae^
SEM		0.369		0.183	0.367
*p* values					
Incubation time		<0.001		<0.001	<0.001
Preplacement holding time		<0.001		<0.001	<0.001
Incubation time × Preplacement holding time		0.978		<0.001	<0.001

^A,B^ Means and percentages in a column within incubation time followed by different letters differ significantly (*p* ≤ 0.05). ^a–e^ Means and percentages in a column within preplacement holding time followed by different letters differ significantly (*p*≤ 0.05).

**Table 4:** Effects of access to feed and water on RYS (g and percentage) of chicks in the 504 and 516 h treatments.

Incubation Time ^1^	Feed and Water Access ^2^	Age at Sampling, h			
		24	48	60	72
		--------------------------------- (g) ---------------------------------			
NIT (504)	Fasted	3.06 ^A^	1.59	1.27	0.76
	No-Fasted	3.20 ^A^	1.51	1.06	0.74
	SEM	0.180	0.115	0.109	0.094
	*p* value ^3^	0.598	0.611	0.173	0.863
LIT (516)	Fasted	2.38 ^B^	1.31	1.02	0.66
	No-Fasted	2.41 ^B^	1.28	0.98	0.70
	SEM	0.141	0.082	0.102	0.059
	*p* value ^3^	0.861	0.852	0.758	0.631
		--------------------------------- (%) ---------------------------------			
NIT (504)	Fasted	7.02 ^A,x^	3.83 ^x^	3.10 ^x^	1.94 ^x^
	No-Fasted	5.88 ^A,y^	2.27 ^y^	1.55 ^y^	0.95 ^y^
	SEM	0.350	0.229	0.197	0.192
	*p* value ^3^	0.024	<0.001	<0.001	0.001
LIT (516)	Fasted	5.62 ^B,x^	3.22 ^x^	2.56 ^x^	1.78 ^x^
	No-Fasted	4.46 ^B,y^	1.99 ^y^	1.39 ^y^	0.89 ^y^
	SEM	0.295	0.169	0.172	0.119
	*p* value ^3^	0.007	<0.001	<0.001	<0.001

^1^ Incubation time of chicks. ^2^ No-fasted group with access to feed and water at 6 h. ^3^ *p* value for comparison of the two groups (fasted/no-fasted; ^x,y^: *p* < 0.03). ^A,B^ Means in each cell followed by different letters differ significantly between incubation time treatments (*p* < 0.05).

**Table 5:** Crop fill progression at 3 h after placement of chicks.

Treatment		Crop Fill Progression at 3 h after Placement of Chicks ^1^				
		Empty	Water	Feed	Full, Rounded	Total
Incubation time (h)		------------------------------------------- (%) -------------------------------------------				
NIT (504)		3.33 ^A^	7.57	6.19	82.90 ^B^	100.0
LIT (516)		0.56 ^B^	6.78	5.22	87.44 ^A^	100.0
SEM		0.343	1.048	0.774	2.231	
Preplacement holding time (h)						
6		8.61 ^a^	15.28 ^a^	14.72 ^a^	61.39 ^c^	100.0
24		0.28 ^b^	8.33 ^b^	5.28 ^b^	86.61 ^b^	100.0
48		0.56 ^b^	3.89 ^b^	5.56 ^b^	90.00 ^ab^	100.0
60		0.28 ^b^	5.04 ^b^	1.59 ^c^	93.10 ^a^	100.0
72		0.00 ^b^	3.33 ^b^	1.39 ^c^	95.28 ^a^	100.0
SEM		0.542	1.658	1.223	1.864	
Incubation time × Preplacement holding time						
NIT (504)	6	15.56 ^Aa^	13.33	16.67	54.44 ^Bc^	100.0
	24	0.00 ^Ab^	7.78	5.00	87.22 ^Aab^	100.0
	48	1.11 ^Ab^	6.11	6.11	86.67 ^Ab^	100.0
	60	0.00 ^Ab^	6.75	2.06	91.19 ^Aab^	100.0
	72	0.00 ^Ab^	3.89	1.11	95.00 ^Aa^	100.0
LIT (516)	6	1.67 ^Ba^	17.22	12.78	68.33 ^Ac^	100.0
	24	0.56 ^Aa^	8.39	5.06	86.00 ^Ab^	100.0
	48	0.00 ^Aa^	1.67	5.00	93.33 ^Aab^	100.0
	60	0.56 ^Aa^	3.33	1.11	95.00 ^Aab^	100.0
	72	0.00 ^Aa^	2.78	1.67	95.56 ^Aa^	100.0
SEM		0.766	2.244	1.730	2.636	
*p* values						
Incubation time		<0.001	0.595	0.380	0.009	
Preplacement holding time		<0.001	<0.001	<0.001	<0.001	
Incubation time × Preplacement holding time		<0.001	0.395	0.699	0.037	

^1^ Crop fill progression divided into the following categories: empty, water only, feed only, and full, soft, and rounded (water and feed). ^A,B^ Percentages of chicks in a column within incubation time followed by different letters differ significantly (*p* ≤ 0.05). ^a–c^ Percentages of chicks in a column within preplacement holding time followed by different letters differ significantly (*p*≤ 0.05).

**Table 6:** Behavior observations of chicks at 1, 3, and 8 h after placement.

Treatment	Observation at 1 h ^1^			Observation at 3 h			Observation at 8 h	
	Eating	Drinking		Eating	Drinking		Eating	Drinking
Incubation time (h)	------------------------------------------- (%) -------------------------------------------							
NIT (504)	23.65	10.06		22.52	10.10		14.02	6.48
LIT (516)	23.79	10.71		23.40	10.90		15.56	7.52
SEM	0.883	0.515		0.662	0.547		0.808	0.580
Preplacement holding time (h)								
6	12.76 ^b^	8.70 ^b^		14.90 ^b^	7.45 ^c^		7.81 ^b^	6.56 ^b^
24	26.15 ^a^	9.70 ^b^		26.20 ^a^	15.47 ^a^		15.63 ^a^	9.90 ^a^
48	25.73 ^a^	10.94 ^ab^		24.27 ^a^	11.56 ^b^		16.35 ^a^	6.46 ^b^
60	28.80 ^a^	12.81 ^a^		26.09 ^a^	9.27 ^bc^		16.15 ^a^	5.37 ^b^
72	25.16 ^a^	9.79 ^b^		23.33 ^a^	8.75 ^c^		18.02 ^a^	6.72 ^b^
SEM	1.396	0.493		1.047	0.865		1.277	0.917
*p* values								
Incubation time	0.907	0.161		0.091	0.225		0.183	0.210
Preplacement holding time	<0.001	0.010		<0.001	<0.001		<0.001	0.014
Incubation time × Preplacement holding time	0.142	0.166		0.481	0.510		0.776	0.348

^1^ Percentage of the behaviors of eating from the feeder and chick paper or drinking water from the nipples at 1 h, 3 h, or 8 h after placement. ^a–c^ Percentages of chicks in a column followed by different letters differ significantly (*p* ≤ 0.05).

**Table 7:** Body weight of chickens from 0 to 7 and 35 d of placement.

Treatment		Body Weight		
		0 d	7 d	35 d
Incubation time		--------------------------- (g) ---------------------------		
NIT (504)		42.16 ^A^	171.6 ^A^	2163
LIT (516)		40.45 ^B^	169.0 ^B^	2162
SEM		0.077	0.47	8.6
Preplacement holding time (h)				
6		44.82 ^a^	176.2 ^a^	2179 ^ab^
24		43.08 ^b^	177.1 ^a^	2202 ^a^
48		41.04 ^c^	170.7 ^b^	2177 ^ab^
60		39.64 ^d^	167.1 ^c^	2148 ^b^
72		37.95 ^e^	160.3 ^d^	2098 ^c^
SEM		0.122	0.74	13.5
Incubation time × Preplacement holding time (h)				
NIT (504)	6	45.68	174.6 ^Bab^	2158
	24	43.96	176.6 ^Aa^	2181
	48	41.85	172.4 ^Abc^	2190
	60	40.61	170.2 ^Ac^	2168
	72	38.70	164.3 ^Ad^	2108
LIT (516)	6	43.95	177.8 ^Aa^	2199
	24	42.19	177.7 ^Aa^	2222
	48	40.23	169.0 ^Bb^	2164
	60	38.68	164.0 ^Bc^	2129
	72	37.19	156.4 ^Bd^	2088
SEM		0.172	1.04	19.1
*p* values				
Incubation time		<0.001	<0.001	0.887
Preplacement holding time		<0.001	<0.001	<0.001
Sex ^1^				<0.001
Incubation time × Preplacement holding time		0.797	<0.001	0.120
Incubation time × Sex				0.530
Preplacement holding time × Sex				0.320
Incubation time × Preplacement holding time × Sex				0.778

^1^ As expected, males exhibited greater BW than females at 35 d (*p* < 0.001), and no interaction effects were observed between sex and other factors (*p* > 0.05). ^A,B^ Means in a column within incubation time followed by different letters differ significantly (*p* ≤ 0.05). ^a–e^ Means in a column within preplacement holding time followed by different letters differ significantly (*p*≤ 0.05).

**Table 8:** Mortality of chickens from placement to 7 and 35 d of placement.

Treatment		Mortality	
		0–7 d	0–35 d
Incubation time		-------------------- (%) --------------------	
NIT (504)		0.94	5.13
LIT (516)		1.04	5.27
SEM		0.197	0.246
Preplacement holding time (h)			
6		0.57 ^b^	4.85 ^b^
24		0.52 ^b^	4.22 ^b^
48		0.89 ^b^	5.37 ^ab^
60		1.15 ^b^	5.42 ^ab^
72		1.82 ^a^	6.15 ^a^
SEM		0.311	0.389
Incubation time × Pre-Placement time (h)			
NIT (504)	6	0.73	5.31
	24	0.52	4.38
	48	0.94	5.10
	60	1.25	5.21
	72	1.25	5.63
LIT (516)	6	0.42	4.38
	24	0.52	4.06
	48	0.83	5.63
	60	1.04	5.63
	72	2.40	6.67
SEM		0.440	0.550
*p* values			
Incubation time		0.710	0.680
Preplacement holding time		0.031	0.028
Incubation time × Preplacement holding time		0.465	0.435

^a,b^ Means in a column with different superscripts differ significantly (*p* ≤ 0.05).

## Data Availability

The data presented in this study are available on request from the corresponding author.

## References

[1] Almeida J.G., Vieira S.L., Gallo B.B., Conde O.R.A., Olmos A.R. (2006). Period of incubation and posthatching holding time influence on broiler performance. Braz. J. Poult. Sci..

[2] Avşar K.O., Uçar A., Özlü S., Elibol O. (2021). Effect of high eggshell temperature during the early period of incubation on hatchability, hatch time, residual yolk and first-week broiler performance. J. Appl. Poult. Res..

[3] Careghi C., Tona K., Onagbesan O., Buyse J., Decuypere E., Bruggeman V. (2005). The effects of the spread of hatch and interaction with delayed feed access after hatch on broiler performance until seven days of age. Poult. Sci..

[4] Decuypere E., Bruggeman V. (2007). The endocrine interface of environmental and egg factors affecting chick quality. Poult. Sci..

[5] Elibol O., Hodgetts B., Brake J. (2002). The effect of storage and pre-warming periods on hatch time and hatchability. Avian Poult. Biol. Rev..

[6] Özlü S., Elibol O. (2023). Rapid egg cooling rate after oviposition influences the embryonic development, hatchability, and hatch time of young and old broiler hatching eggs. Poult. Sci..

[7] Tona K., Bamelis F., De Ketelaere B., Bruggeman V., Moraes V.M.B., Buyse J., Onagbesan O., Decuypere E. (2003). Effects of egg storage time on spread of hatch, chick quality, and chick juvenile growth. Poult. Sci..

[8] Aviagen Factors Affecting Chick Comfort and Liveability from Hatcher to Brooding House. https://www.thepoultr-ysite.com/articles/factors-affecting-chick-comfort-and-liveability-from-hatcher-to-brooding-house.

[9] Freeman B.M. (1984). Transportation of poultry. World’s Poult. Sci. J..

[10] Peebles E.D., Barbosa T.M., Cummings T.S., Dickson J., Womack S.K. (2016). Comparative effects of in ovo versus subcutaneous administration of the Marek’s disease vaccine and pre-placement holding time on the early post-hatch quality of Ross × Ross 708 broiler chicks. Poult. Sci..

[11] Dibner J.J., Knight C.D. (1999). Early feeding and gut health in hatchlings. Int. Hatchery Pract..

[12] Hamdy A.M.M., Henken A.M., Van der Hel W., Galal A.G., Abd-Elmoty A.K.I. (1991). Effects of incubation humidity and hatching time on heat tolerance of neonatal chicks: Growth performance after heat exposure. Poult. Sci..

[13] Vieira S., Moran E. (1999). Effects of delayed placement and used litter on broiler yields. J. Appl. Poult. Res..

[14] Fanguy R.C., Misra L.K., Vo K.V., Blohowiak C.C., Krueger W.F. (1980). Effect of delayed placement on mortality and growth performance of commercial broilers. Poult. Sci..

[15] Hager J.E., Beane W.L. (1983). Posthatch incubation time and early growth of broiler chickens. Poult. Sci..

[16] Sklan D., Noy Y., Hoyzman A., Rozenboim I. (2000). Decreasing weight loss in the hatchery by feeding chicks and poults in hatching trays. J. Appl. Poult. Res..

[17] Casteel E., Wilson J., Buhr R., Sander J. (1994). The influence of extended posthatch holding time and placement density on broiler performance. Poult. Sci..

[18] Joseph N.S., Moran E.T. (2005). Effect of flock age and postemergent holding in the hatcher on broiler live performance and further-processing yield. J. Appl. Poult. Res..

[19] Lamot D.M., van de Linde I.B., Molenaar R., van der Pol C.W., Wijtten P.J., Kemp B., van den Brand H. (2014). Effects of moment of hatch and feed access on chicken development. Poult. Sci..

[20] Özlü S., Shiranjang R., Elibol O., Brake J. (2018). Effect of hatching time on yolk sac percentage and broiler live performance. Braz. J. Poult. Sci..

[21] Özlü S., Uçar A., Romanini C.E.B., Banwell R., Elibol O. (2020). Effect of posthatch feed and water access time on residual yolk and broiler live performance. Poult. Sci..

[22] Stamps L.K., Andrews L.D. (1995). Effects of delayed housing of broiler chicks and three different types of waterers on broiler performance. Poult. Sci..

[23] Özlü S., Erkuş T., Kamanlı S., Nicholson A.D., Elibol O. (2022). Influence of the preplacement holding time and feeding hydration supplementation before placement on yolk sac utilization, the crop filling rate, feeding behavior and first-week broiler performance. Poult. Sci..

[24] Nielsen S.S., Alvarez J., Bicout D.J., Calistri P., Canali E., Drewe J.A., Garin-Bastuji B., Gonzales Rojas J.L., Gortázar Schmidt C., EFSA Panel on Animal Health and Welfare (AHAW) (2022). Welfare of domestic birds and rabbits transported in containers. EFSA J..

[25] Aviagen Ross Broilers Nutrition Specifications. http://en.aviagen.com/assets/TechCenter/RossBroiler/Ross-BroilerNutritionSpecifications2022-EN.pdf.

[26] Decuypere E., Tona K., Bruggeman V., Bamelis F. (2001). The day-old chick: A crucial hinge between breeders and broilers. World’s Poult. Sci. J..

[27] Hamissou Maman A., Özlü S., Uçar A., Elibol O. (2019). Effect of chick body temperature during post-hatch handling on broiler live performance. Poult. Sci..

[28] French N.A., Nicholson D.A., Kretzschmarb V., Goyne D. Effect of post-hatch holding temperature on chick behaviour, gut development and broiler performance. Proceedings of the Incubation and Fertility Research Group Meeting.

[29] Lourens S., Holleman J., van Schie T. (2021). Hatchery Signals: A Practical Guide to Improving Hatching Results.

[30] Mitchell M.A., Kettlewell P.J., Weeks C.A., Butterworth A. (2004). Transport and handling. Measuring and Auditing Broiler Welfare.

[31] Mitchell M.A., Kettlewell P.J. (2004). Transport of chicks, pullets and spent hens. Welfare of the Laying Hen, Proceedings of the 27th Poultry Science Symposium of the World’s Poultry Science Association (UK Branch), Bristol, UK, 17–20 July 2003.

[32] Yerpes M., Llonch P., Manteca X. (2021). Effect of environmental conditions during transport on chick weight loss and mortality. Poult. Sci..

[33] EFSA (European Food Safety Authority) Panel on Animal Health and Welfare (2011). Scientific Opinion concerning the welfare of animals during transport. EFSA J..

[34] Van der Wagt I., de Jong I.C., Malcolm M.A., Molenaar R., van den Brand H. (2020). A review on yolk sac utilization in poultry. Poult. Sci..

[35] Deines J.R., Clark F.D., Yoho D.E., Bramwell R.K., Rochell S.J. (2021). Effects of hatch window and nutrient access in the hatcher on performance and processing yield of broiler chicks reared according to time of hatch. Poult. Sci..

[36] Gonzales E., Kondo N., Saldanha E., Loddy M., Careghi C., Decuypere E. (2003). Performance and physiological parameters of broiler chickens subjected to fasting on the neonatal period. Poult. Sci..

[37] Franco J.R.G., Murakami A.E., Natali M.R.M., Garcia E.R.M., Furlan A.C. (2006). Influence of delayed placement and dietary lysine levels on small intestine morphometrics and performance of broilers. Braz. J. Poult. Sci..

[38] Van den Brand H., Molenaar R., Van der Star I., Meijerhof R. (2010). Early feeding affects resistance against cold exposure in young broiler chickens. Poult. Sci..

[39] Aviagen Ross Broiler Management Handbook. http://tmea.aviagen.com/assets/TechCenter/RossBroiler/Ross-BroilerHandbook2018-EN.pdf.

[40] Boyner M., Ivarsson E., Franko M.A., Rezaei M., Wall H. (2021). Effect of hatching time on time to first feed intake, organ development, enzymatic activity and growth in broiler chicks hatched on-farm. Animal.

[41] Hollemans M.S., de Vries S., Lammers A., Clouard C. (2018). Effects of early nutrition and transport of 1-day-old chickens on production performance and fear response. Poult. Sci..

[42] Baião N.C., Cançado S.V., Lúcio C.G. (1998). Effect of hatching period and the interval between hatching and housing on broiler performance. Arw. Bras. Med. Vet. Zootec.

[43] Bergoug H., Guinebretière M., Tong Q., Roulston N., Romanini C.E.B., Exadaktylos V., Berckmans D., Garain P., Demmers T.G.M., McGonnell I.M. (2013). Effect of transportation duration of 1-day-old chicks on postplacement production performances and pododermatitis of broilers up to slaughter age. Poult. Sci..

[44] Boyner M., Ivarsson E., Wattrang E., Sun L., Wistedt A., Wall H. (2023). Effects of access to feed, water, and a competitive exclusion product in the hatcher on some immune traits and gut development in broiler chickens. Br. Poult. Sci..

[45] Cengiz O., Koksal B.H., Tatli O., Sevim O., Avci H., Epikmen T., Beyaz D., Buyukyoruk S., Boyacioglu M., Uner A. (2012). Influence of dietary organic acid blend supplementation and interaction with delayed feed access after hatch on broiler growth performance and intestinal health. Vet. Med..

[46] Fairchild B.D., Northcutt J.K., Mauldin J.M., Buhr R.J., Richardson L.J., Cox N.A. (2006). Influence of water provision to chicks before placement and effects on performance and incidence of unabsorbed yolk sacs. J. Appl. Poult. Res..

[47] Li D.L., Wang J.S., Liu L.J., Li K., Xu Y.B., Ding X.Q., Wang Y.Y., Zhang Y.F., Xie L.Y., Liang S. (2022). Effects of early post-hatch feeding on the growth performance, hormone secretion, intestinal morphology, and intestinal microbiota structure in broilers. Poult. Sci..

[48] Wang Y., Li Y., Willems E., Willemsen H., Franssens L., Koppenol A., Guo X., Tona K., Decuypere E., Buyse J. (2014). Spread of hatch and delayed feed access affect post hatch performance of female broiler chicks up to day 5. Animal.

[49] Dişa R., Özlü S., Elibol O. (2022). Research Note: Interaction between hatching time and chick pull time affects broiler live performance. Poult. Sci..

[50] Cardeal P.C., Rocha J.S.R., Pompeu M.A., Pereira L.F.P., Saldanha M.M., Baiao N.C., Araujo I.C.S., Lara L.J.C. (2020). Effects of placement time on performance and gastrointestinal tract growth of male broiler chickens. Braz. J. Anim. Sci..

[51] De Jong I.C., Van Riel J., Lourens A., Bracke M.B.M., Van den Brand H. (2016). Effects of food and water deprivation in newly hatched chickens. A systematic literature review and meta-analysis. Wageningen Livestock Research.

